# 2420. Analysis of Diagnostic Criteria for ECMO-Associated Pneumonia

**DOI:** 10.1093/ofid/ofad500.2040

**Published:** 2023-11-27

**Authors:** Julie England, Rongbing Xie, Tammy Marshall, James Kirklin, Peggy Blood, Keith Wille, Enrique Gongora, Brandon Sharp, Anoma Nellore, Rachael A Lee, Jeremey Walker

**Affiliations:** UAB Medical Center, Birmingham, Alabama; UAB Medical Center, Birmingham, Alabama; UAB Medical Center, Birmingham, Alabama; UAB Medical Center, Birmingham, Alabama; UAB Medical Center, Birmingham, Alabama; UAB Medical Center, Birmingham, Alabama; UAB Medical Center, Birmingham, Alabama; UAB Medical Center, Birmingham, Alabama; University of Alabama Birmingham, Birmingham, AL; University of Alabama at Birmingham, Birmingham, AL; University of Alabama Birmingham, Birmingham, AL

## Abstract

**Background:**

Ventilator-associated pneumonia (VAP) is a well-established cause of morbidity and mortality in critically ill patients, but diagnostic criteria exclude patients on extracorporeal membrane oxygenation (ECMO). Despite this, VAP has been used to approximate pneumonia in patients on ECMO for study purposes (Figure 1). An adequate definition to recognize and prevent nosocomial pneumonia in this population is needed. Our institution began looking at a set of diagnostic criteria for EAP (ECMO-Associated Pneumonia, Figure 2). We performed a retrospective analysis to test the validity of VAP and our new EAP definition as diagnostic criteria for pneumonia in ECMO patients.

**Figure 1.**
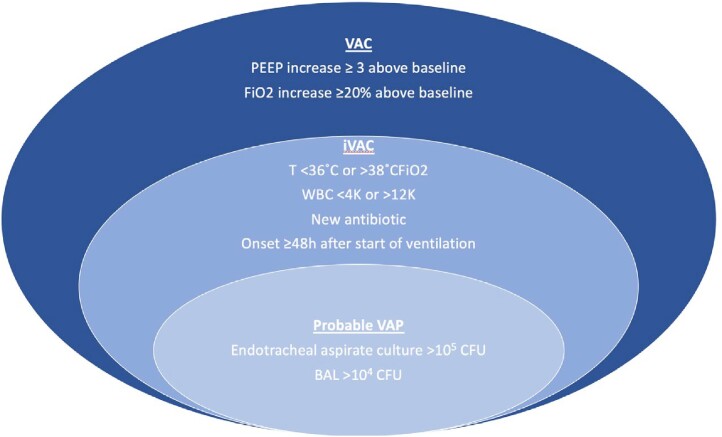
Criteria for Probable VAP. Simplified from NHSN criteria. VAC=ventilator-associated condition. iVAC=infection-related ventilator-associated condition. VAP=ventilator-associated pneumonia.

**Figure 2.**
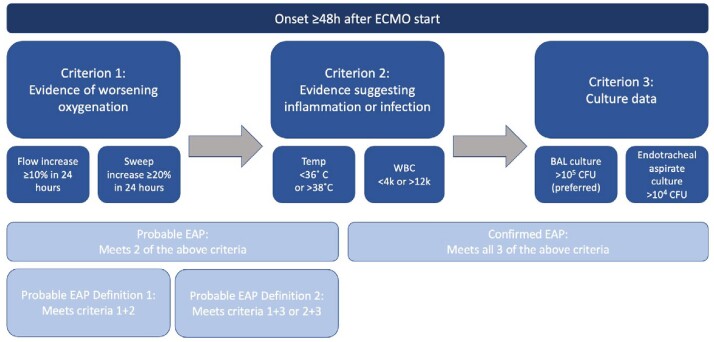
Proposed diagnostic criteria for ECMO-associated pneumonia (EAP)

**Methods:**

Our center maintains a database of clinical parameters for patients on ECMO, which includes outcomes of interest verified in real time. Patient charts coded as having an outcome of pneumonia from 2018 to 2021 were pulled and independently adjudicated to determine if they met our inclusion criteria (Figure 3). 20 patients were identified as having at least one event. We observed their stay on ECMO up to 30 days. We calculated the established VAP criteria (Figure 1) and our proposed EAP criteria (Figure 2) daily. Dates of clinical pneumonia were time of positive culture and subsequent 5 days.

**Figure 3.**
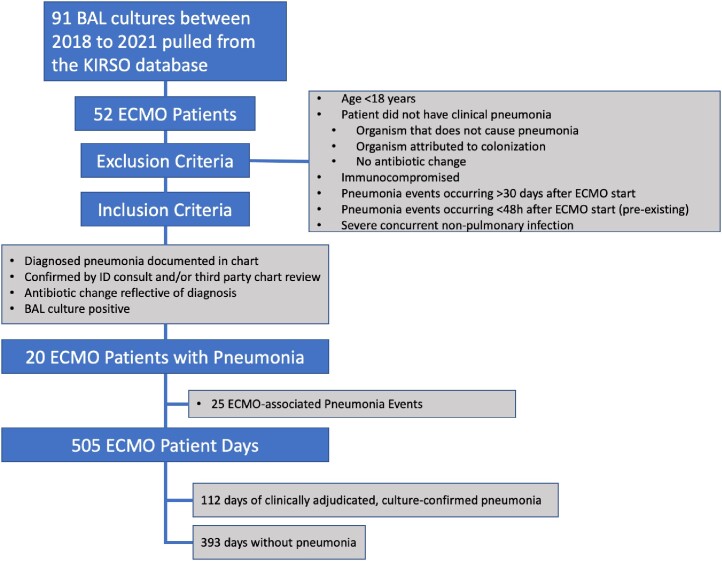
Selection process for study population. BAL=bronchoalveolar lavage

**Results:**

85% of patients were on VV ECMO and 50% patients with SARS-CoV-2 (Table 1). In 20 patients with clinically diagnosed, culture confirmed pneumonia, only 1% of patients met criteria for Probable VAP (sensitivity 4%, Table 2). Probable EAP Definition 2 was the most sensitive model for predicting clinical pneumonia (sensitivity 91.1%), followed distantly by the Confirmed EAP model (sensitivity 19.6%). The EAP model had a stronger relationship with clinical pneumonia than the VAP model as demonstrated by the ROC curves (Figure 4). Individual components (flow, sweep, FiO2, PEEP) could not predict onset of clinical pneumonia within 48 to 72 hours.

**Table 1.**
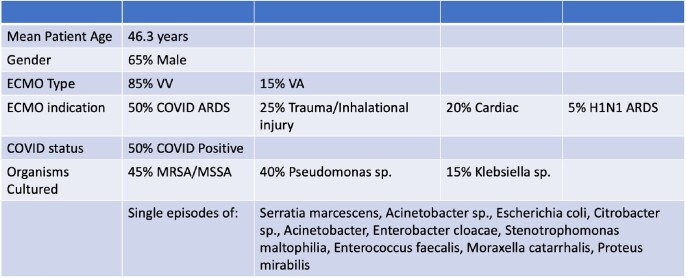
Characteristics of study population

Table 2.

**Figure:**
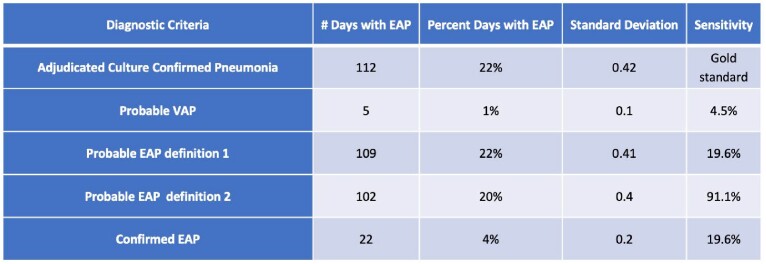

Percentage of days with pneumonia as identified by each set of diagnostic criteria, with sensitivity calculated.

Figure 4

**Figure:**
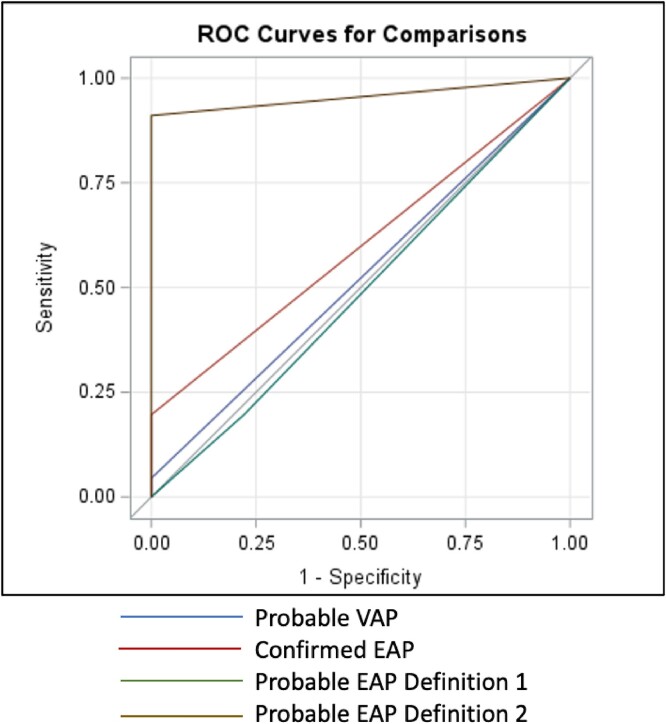

ROC curves for comparison. Probable EAP Definition 2 had the highest sensitivity for diagnosing EAP.

**Conclusion:**

Our study reiterates that VAP criteria should not be used to approximate diagnosis of pneumonia in patients on ECMO. As oxygenation and sepsis triggers adjustment of flow and sweep we found this in combination with infectious parameters, and BAL culture that was more sensitive in predicting clinical pneumonia. These measures require validation in a larger cohort.

**Disclosures:**

**James Kirklin, MD**, Data Center for STS Intemacs/Pedimacs Registries: Director, partial salary support|Kirklin Solutions, Inc.: Ownership Interest

